# Adolescent Health and High School Dropout: A Prospective Cohort Study of 9000 Norwegian Adolescents (The Young-HUNT)

**DOI:** 10.1371/journal.pone.0074954

**Published:** 2013-09-23

**Authors:** Karin A. A. De Ridder, Kristine Pape, Roar Johnsen, Turid Lingaas Holmen, Steinar Westin, Johan Håkon Bjørngaard

**Affiliations:** 1 Department of Public Health and General Practice, Norwegian University of Science and Technology, Trondheim, Norway; 2 Department of Physical Medicine and Rehabilitation, Levanger Hospital, Nord-Trøndelag Health Trust, Levanger, Norway; 3 HUNT Research Center, Department of Public Health and General Practice, Norwegian University of Science and Technology, Levanger, Norway; 4 Forensic Department and Research Centre Bröset, St. Olavs Hospital, Trondheim University Hospital, Trondheim, Norway; Hunter College, City University of New York (CUNY), CUNY School of Public Health, United States of America

## Abstract

**Background:**

High school dropout is of major concern in the western world. Our aims were to estimate the risk of school dropout in adolescents following chronic somatic disease, somatic symptoms, psychological distress, concentration difficulties, insomnia or overweight and to assess to which extent the family contributes to the association between health and school dropout.

**Methods:**

A population of 8950 school-attending adolescents (13–21 years) rated their health in the Young-HUNT 1 Study (90% response rate) in 1995–1997. High school dropout or completion, was defined with the Norwegian National Education Database in the calendar year the participant turned 24 years old. Parental socioeconomic status was defined by using linkages to the National Education Database, the National Insurance Administration and the HUNT2 Survey. We used logistic regression to estimate odds ratios and risk differences of high school dropout, both in the whole population and among siblings within families differentially exposed to health problems.

**Results:**

All explored health dimensions were strongly associated with high school dropout. In models adjusted for parental socioeconomic status, the risk differences of school dropout according to health exposures varied between 3.6% (95% CI 1.7 to 5.5) for having ≥1 somatic disease versus none and 11.7% (6.3 to 17.0) for being obese versus normal weight. The results from the analyses comparing differentially exposed siblings, confirmed these results with the exception of weaker associations for somatic diseases and psychological distress. School dropout was strongly clustered within families (family level conditional intraclass correlation 0.42).

**Conclusions:**

Adolescent health problems are markers for high school dropout, independent of parental socioeconomic status. Although school dropout it strongly related to family-level factors, also siblings with poor health have reduced opportunity to complete high school compared to healthy siblings. Public health policy should focus on ensuring young people with poor health the best attainable education.

## Introduction

High school dropout is a major concern in most Western countries because it is associated with lower employment rate and poor health [1], [2]. Already in the first decade of adulthood, school dropout is associated with a substantially higher probability of receiving medical and non-medical social insurance benefits, suggesting that mechanisms in adolescence are at the basis of these adversities [3].

Adult health is strongly related to educational attainment. 4While prior research has mainly considered poor health a consequence of low education, recent twin studies have suggested that, in some cases, the relation can be the result of health selection – poor health causing lower education [4], [5]. There is evidence that suggests an association between poor health in adolescence and low educational achievement, as self-rated health in adolescence is associated with adult educational level [6], [7]. Other studies have indicated that chronic physical conditions or disabilities [8], mental or psychosomatic symptoms [9], attention problems [10], and sleep problems [11] are associated with poor educational attainment. In addition, height and weight, which reflects a latent health potential, results in differences in educational attainment [12].

However, confounding from familial genetic, environmental and socioeconomic factors could also influence these associations [13], yet it is still unclear to what extent family factors can be attributed to the association between health and school dropout.

We studied the associations between several dimensions of self-reported health in adolescence and high school dropout, adjusting for parental socioeconomic background and family living situation. Additionally, by comparing siblings, we tested if the associations remained after accounting for all shared, stable unobserved family characteristics.

## Methods

### Participants

The Young-Hunt study is the adolescent part of the HUNT study (The Nord-Trøndelag Health Study, http://www.ntnu.no/hunt) in the county Nord-Trøndelag, Norway [14]. All school-attending students in the middle and secondary school were invited to fill in a comprehensive questionnaire during a class hour, and 8949 completed the questionnaire (90% response rate). This population based survey was carried out between autumn 1995 and spring 1997. Participants were linked to their biological parents through the National Identity Number. Adolescents and their parents were linked to the Norwegian National Education Database (http://www.ssb.no/mikrodata). Parental information was also obtained by linkage to the National Insurance Administration (income) and the HUNT2 study (occupational class). Siblings (having the same biological mother) were identified through the National Register Code in the family register. We excluded 76 individuals because of missing educational data (8), age-school mismatch (4), born after 1983 (4), died during follow-up (30) and disability pension within the period (16–21 years) when they were eligible for high school education (30).

The Regional Committee for Medical Research Ethics approved the present study (reference 2010/1527-5, in accordance with the Helsinki declaration). Each participant and the parents/legal guardians of the participants younger than 16 years old gave their written consent to participate in the Young-Hunt Study.

### School dropout

In Norway, basic education is compulsory up to the start of senior high school (upper secondary education) at age 16. Every 15- to 16-year-old has a statutory right to 3 years of senior high school which consists of both general and vocational tracks. In the follow-up period (1998-2008), we registered the outcome high school for all participants as either completion or dropout in the calendar year the participant turned 24 years old. We accomplished this using the linkage to the Norwegian National Education Database.

### Health measures

We identified several health dimensions based on the self-reported health information provided by the study participants. We defined somatic disease as having asthma, diabetes, migraine, or epilepsy diagnosed by a doctor or having any other illness that lasted longer than 3 months. Subjective health problems are common in adolescence, tend to occur in a cluster and symptom load scores have been considered as measuring a latent trait of psychosomatic complaints [15]. Somatic symptom scores were based on the sum of self-reported presence of eight symptoms (headache, neck or shoulder pain, joint or muscle pain, stomach pain, nausea, constipation, diarrhea, heart palpitations; each one dichotomized into “never/seldom” and “sometimes/often”) during the last 12 months (Cronbachs alpha 0.73). This symptom score was dichotomized into the two lowest tertiles (none or one symptom) versus the highest tertile (two or more symptoms). Psychological distress was measured with the SCL-5 scale score – a validated 4-integer 5 item short version of the original SCL-90 (Hopkins Symptom Checklist) [16]. The variable was dichotomized with a cut-off point at 2.0 [17]. Insomnia was defined by having difficulties falling asleep in the last month and dichotomized into “never/sometimes” versus “often/almost every night”. Concentration difficulties were defined as having difficulties concentrating during class and dichotomized into “never/sometimes” versus “often/very often”. We measured self-rated health using the question “How is your health at the moment?” and dichotomized the four response alternatives into “good/very good” versus “poor/not so good”.

Trained nurses measured height and weight following a standard protocol using standardized meter bands and weight scales. Body mass index (BMI) was defined by cut-offs for the appropriate age groups as proposed by the International Obesity Task Force (IOFT) described by Cole et al. [18] Overweight corresponded with the adult BMI from 25 to 30 and obesity a BMI of 30 and more.

### Parental socio-economic position

Parental education level was registered at the time the participant was 16 years old and divided into three categories: compulsory (primary and lower secondary education), intermediate (upper secondary and post-secondary non-tertiary education) and tertiary (under-graduate, graduate and post-graduate education). Parental income was assessed by the mean annual income (Norwegian currency) in a two year period (1994 and 1995). The total income (including income from benefits) was used and defined by quintiles. Parental occupational class was defined by Erikson Goldthorpe Portocarero (EGP) social class scheme in HUNT2 [19]. The family living situation was defined by living in a “traditional family” (with both the biological mother and father) or not.

### Statistical analysis

Primary analysis investigated the association for each health variable with high school dropout. Sex- and age-adjusted logistic regression analyses were conducted on complete datasets for each model defined by the health variable, with the total N varying for each model. The percentage of missing data varied from 2.0% to 7.5%. To adjust for possible socioeconomic confounders, maternal education level and family living situation were added to the model. Maternal education level was chosen because this measure of parental socioeconomic status (SES) had little missing data (0.5%) compared with the other measures, and 87% of the adolescents were living with their mother. We performed additional analyses using various socioeconomic variables (maternal, paternal and highest parental education, income and occupation separately and combined). We carried out tests for statistical interaction between our health variables and sex and between health variables and parental socioeconomic status. We also performed sensitivity analysis by use of complete case-only (n = 7730), which restricted the analysis to participants with complete data for all exposures, outcomes and confounder variables.

Secondary analysis estimated multivariable sibling fixed-effect (conditional logit) models in order to account for unobserved heterogeneity at the family level (number of siblings = 698). Sibling fixed-effect logistic regression models or logistic regression models conditional on sharing the same biological mother are equivalent [20]. The model attends to the family of origin and focuses on the siblings discordant on high school graduation status. It compares health among siblings within the same families, thereby controlling for all family background characteristics (observed and unobserved) that the siblings share. These sibling fixed-effect models were adjusted for sex, age and family living situation.

Finally, we investigated to what degree high school dropout was determined by the family of origin with sex- and age-adjusted multilevel logistic regression for complete cases (n = 7730). Thereafter, we included the individual characteristics (health variables) and the family variables (maternal education level and family living situation) to investigate the extent to which family level differences were explained by these individual and contextual characteristics. We estimated a conditional intraclass correlation coefficient (ICC) with linear threshold method and the median odds ratio (MOR) [21]. The ICC expresses the propensity to dropout of school that can be attributed to the family. The MOR quantifies the variation between clusters (families) by comparing two persons with the same covariates when randomly chosen from two different families. The MOR is defined as the median odds ratio between the person of higher propensity and the person of lower propensity. If the MOR is one, there is no variation between families.

Point estimates obtained from logistic regression analyses are presented as odds ratios (OR) and risk differences (RD) with 95% confidence intervals (CI). A risk difference describes how 1 unit change in an independent variable (eg, somatic disease or not) alters the absolute risk of a current outcome (eg, high school dropout). Risk differences were estimated from the logistic regression models with the covariates at their mean. Data were analyzed with STATA 12.1 (StataCorp LP).

## Results

### Description of participants

The characteristics of the whole population and the siblings are presented in table 1. The mean follow-up time was 8.0 years (range 3 to 12 years). The baseline mean age of the participants was 16 years (range 13 to 21 years). At the age of 24, 1488 (17%) had not completed high school, more boys (20%) than girls (14%). Compared with the whole sibling sample, the sample of siblings discordant on graduation status (n = 698) was characterized by more mothers with only primary education, fewer traditional families, more psychological distress, poorer self-reported health and more concentration problems.

**Table 1:** Characteristics of the total cohort, all the siblings within the cohort and the siblings with different outcome (school completion/dropout) within the sibling cohort.

	Total cohort	All siblings	Siblings with different outcome
High school dropout	1488 (17)	516 (16)	346 (50)
Mean (SD) Age (years)	16.0 (1.94)	16.1 (2.0)	16.1 (2.1)
Male	4463 (50)	1628 (50)	330 (53)
**Individual health factors**			
Somatic disease			
1 or more	1813 (20)	676 (21)	160 (22)
Missing	(0)	(0)	(0)
Somatic symptoms			
2 or more	3094 (35)	1118 (35)	272 (39)
Missing	(4)	(3)	(5)
Psychological distress			
High	879 (10)	324 (10)	88 (13)
Missing	(2)	(2)	(4)
Insomnia			
Often/every night	887 (10)	316 (10)	81 (12)
Missing	(1)	(1)	(2)
Concentration difficulties			
Often/very often	2101 (24)	771 (24)	215 (31)
Missing	(2)	(2)	(4)
Self-rated health			
Not so good/bad	951 (11)	332 (10)	97 (14)
Missing	(2)	(2)	(3)
BMI			
Overweight	1184 (13)	412 (13)	92 (13)
Obese	251 (3)	84 (3)	22 (3)
Missing	(6)	(6)	(7)
**Family factors**			
Maternal education level			
Primary	2405 (27)	835 (25.5)	275 (39)
Intermediate	4404 (49.5)	1558 (48)	319 (46)
Tertiary	2023 (23)	848 (26)	98 (14)
Missing	(0.5)	(0.5)	(1)
Family living situation			
Traditional family	6418 (74)	2483 (76)	446 (64)
Missing	(2)	(2)	(2)
**Observations**	**8873**	**3256**	**698**

Figures are numbers (percentages), unless stated otherwise.

### Whole study population analyses

There were crude associations between all health variables and a subsequent risk of high school dropout (table 2). The associations were attenuated with adjustment for maternal education level and family living situation. Adjustment for other parental socioeconomic measures (educational level of both parents, parental income and parental occupational class) **–** separately and combined **–** did not alter the results (data not shown). Parental education level was the most important socioeconomic measure and was strongly associated with high school dropout. The absolute increase in the risk of high school dropout according to the different health measures varied between 3.6% (95% CI 1.7 to 5.5) for having 1 or more somatic disease and 11.7% (6.3 to 17.0) for being obese corresponding to the adjusted models in table 3. The risk differences for all variables for the whole population analyses in table 2 are shown in table 3. We performed additional analyses with insomnia, concentration problems and self-rated health as categorical measures (using all 4 categories) and with symptom load and psychological distress as continuous measures. We found indications of a dose-response relationship between all the health variables and the risk for high school dropout. Complete case analyses of only participants with complete data (n = 7730) showed the same associations between the health variables and school dropout. For all health variables, there was no evidence for effect measure modification by sex or maternal education.

**Table 2:** Odds ratio for high school dropout according to indicators of adolescent health in the whole population (crude and adjusted models) and within the families (sibling fixed-effect models).

	Crude^a^		Adjusted^b^	Within family effect^c^	
	N dropout	Odds ratio (CI)	Odds ratio (CI)	N dropout	Odds ratio (CI)
**Somatic disease**					
None	1070	1.00	1.00	244	1.00
1 or more	358	1.39 (1.22 to 1.59)	1.32 (1.15 to 1.51)	78	1.06 (0.70 to 1.60)
**Somatic symptoms**					
None or 1	803	1.00	1.00	169	1.00
2 or more	566	1.51 (1.34 to 1.71)	1.42 (1.25 to 1.62)	125	1.29 (0.87 to 1.90)
**Psychological distress**					
Low	1202	1.00	1.00	298	1.00
High	180	1.69 (1.41 to 2.03)	1.56 (1.30 to 1.88)	37	1.07 (0.64 to 1.78)
**Insomnia**					
Never/seldom	1201	1.00	1.00	272	1.00
Often/every night	193	1.67 (1.40 to 1.99)	1.66 (1.39 to 1.99)	39	1.27 (0.75 to 2.15)
**Concentration difficulties**					
Never/seldom	881	1.00	1.00	197	1.00
Often/very often	497	2.13 (1.88 to 2.43)	1.98 (1.74 to 2.26)	108	1.69 (1.12 to 2.53)
**Self-rated health**					
Very good/good	1163	1.00	1.00	261	1.00
Not so good/bad	245	2.07 (1.77 to 2.43)	1.81 (1.53 to 2.13)	48	1.44 (0.87 to 2.39)
**BMI**					
Normal weight	975	1.00	1.00	133	1.00
Overweight	231	1.47 (1.25 to 1.73)	1.34 (1.14 to 1.58)	39	0.93 (0.55 to 1.56)
Obese	71	2.39 (1.80 to 3.18)	2.20 (1.64 to 2.95)	14	4.18 (1.11 to 15.7)

Values in parentheses are 95% confidence intervals (CI).

a Crude models adjusted for sex and age.

b Adjusted for sex, age, maternal education level and family living situation.

c Sibling fixed-effect models are adjusted for sex, age and family living situation

Total N varies for each health variable in the total population from 8205 to 8696, and in the sibling fixed-effect models from 581 to 649.

**Table 3:** Risk difference (RD) of school dropout from logistic regression models^a^.

	Crude^b^	Adjusted^c^
	Risk difference (CI)	Risk difference (CI)
**1 or more somatic disease**	4.8 (2.8 to 6.9)	3.6 (1.7 to 5.5)
Versus none	ref.	ref.
**2 or more somatic symptoms**	5.7 (4.0 to 7.5)	4.5 (2.8 to 6.2)
Versus none or 1	ref.	ref.
**High psychological distress**	8.0 (4.9 to 11.1)	6.2 (3.3 to 9.0)
Versus low	ref.	ref.
**Often/every night insomnia**	7.8 (4.8 to 10.8)	7.2 (4.3 to 10.0)
Versus Never/seldom	ref.	ref.
**Often/very often concentration difficulties**	11.3 (9.2 to 13.4)	9.3 (7.4 to 11.3)
Versus never/seldom	ref.	ref.
**Not so good/bad self-rated health**	11.7 (8.7 to 14.7)	8.5 (5.8 to 11.3)
Versus very good/good	ref.	ref.
**BMI**		
Normal weight	ref.	ref.
Overweight	5.4 (3.0 to 7.8)	3.7 (1.5 to 5.9)
Obese	14.1 (8.4 to 19.7)	11.7 (6.3 to 17.0)

Figures are percentages with 95% confidence interval (CI).

a Estimated risk difference in the risk to drop out of high school relative to complete high school.

b Crude models with the covariates sex and age at mean.

c Adjusted models with the covariates sex, age, maternal education level and family living situation at mean

Total N varies for each health variable from 8205 to 8696.

### Within family analyses

The sibling fixed-effect analysis confirmed the results from the total population, except for somatic diseases and psychological distress; although the precision was reduced due to reduced statistical power in the within-family models (table 2).

### Clustering by family analyses

High school drop-out was substantially clustered in families (table 4). About 42% of the adolescents’ propensity to drop out of high school could be attributed to the family. Likewise, the median of the odds ratios (MOR) between the person with a high propensity and the person with a low propensity is estimated to be 4.3. When individual health variables and contextual factors (maternal education level and family living situation) were included, the unexplained cluster heterogeneity decreased substantially, yielding a MOR of 2.98. However, a large proportion of the clustering by family still remained unexplained.

**Table 4:** Clustering of high school dropout on the family level for complete cases (n = 7730).

	Crude^a^	Adjusted^b^ for health	Adjusted^c^ for health and family characteristics
**ICC**	41.8%	36.4%	28.7%
**MOR**	4.30	3.68	2.98

Figures are intraclass coefficients (ICC%) and median odds ratios (MOR).

a Crude model adjusted for sex and age.

b Model adjusted for sex, age, somatic disease, somatic symptoms, psychological distress, insomnia, concentration difficulties, self-rated health and BMI.

c Model adjusted for sex, age, somatic disease, somatic symptoms, psychological distress, insomnia, concentration difficulties, self-rated health, BMI, maternal education level and family living situation.

## Discussion

In this large prospective population based study over 11 years, we found an increased risk of high school dropout for all explored dimensions of adolescent ill health. With the exception of psychological distress and somatic disease, this was also true when comparing siblings. Although high school dropout was strongly associated with parental socioeconomic class and strongly clustered at the family level, the negative impact of ill health on school dropout seemed to exist in all families and across all social classes.

### Strengths and limitations of the study

The results were based on a large number of participants, and outcome measures were attained from nearly complete and accurate register-based information. Furthermore, we were able to control for several confounding variables, and our sibling design made it possible to control for any known and unknown family factors shared by siblings. Although the participation rate was high (90%), we cannot rule out the possibility of selection bias: firstly, it is reasonable to assume higher dropout rates among the non-responders and secondly, we included only the adolescents enrolled in school at baseline, which may have excluded especially older adolescents who had already dropped out from school. Selection bias might affect the results with attenuated associations between adolescent health problems and school dropout. We relied mainly on self-reported health measurements, and it is noteworthy that structured clinical assessments of the participants’ health status could have given more valid and reliable baseline information. However, such an approach would not have been feasible in a study of this size. The precision of the sibling fixed-effect analyses was reduced due to the lower number of siblings compared to the whole population.

### Comparison with other studies

The literature related to adolescent health and educational attainment is limited, and studies with a prospective design have been sparse. On the population level, we found dose-response associations between psychological distress, somatic disease, symptom load, insomnia, concentration difficulties, self-rated health and overweight/obesity and school dropout, which is consistent with other studies [8], [11], [22]–[32]. The effect of obesity on school dropout is greater than other health problems, which is consistent with Gortmaker et al. [31].

In cases where there was a high symptom load, insomnia, concentration difficulties, poor self-rated health and obesity, the impact of poor adolescent health remained even when controlling for stable family background characteristics. In the case of self-rated health, this finding is consistent with other studies [29], [33], but for concentration difficulties, this finding is inconsistent with the study of Fletcher et al. [28] in that they did not find any effect of ADHD symptoms on risk for high school dropout within the family. However, a self-report of concentration difficulties is not the same as a screening set for ADHD symptoms, and there is some evidence that inattention rather than hyperactivity predicts low long-term educational attainment [34]. We are not aware of other studies that have compared siblings with different levels of symptom load, insomnia, or weight and the risk of subsequent school dropout. Our results strengthen the hypothesis that health problems in adolescence could have adverse causal effect on future socioeconomic position.

The results of the within-family models differ from the whole-population models for somatic disease, psychological distress and overweight. Their impact on school dropout was completely attenuated when comparing siblings differentially exposed. This may reflect the confounding effect of shared family background characteristics and suggests that such shared factors are essential in the association between health and school dropout. However, psychological distress is also clustered within families [35], and an on-off measure of symptoms of mental illness may not be enough to differentiate psychiatric pathology between siblings. Fletcher et al. [36] found that siblings with depression had a higher risk for dropout compared to their siblings without depression. Also, Fletcher and Richards [26] found lower educational attainment for adolescents with diabetes. Our variable on somatic disease included diabetes, but we could not reproduce the same analyses with only diabetes because of lack of power.

As in many other studies, adolescents from lower socioeconomic classes had substantial higher risk for not completing high school [37]. Our study showed that *all* examined health dimensions increased the risk for school dropout independent of, and additive to, socioeconomic group defined by parental education, income or occupation, which is in concordance with other studies [29], [30], [38]. The relationship between poor health and school dropout was not less important for higher social classes. Previous research suggested that socioeconomic inequalities in health during adult life were to a large extent due to social causation, and health selection was only slightly involved [39], [40]. However, most of this research was on adult populations. Our study suggests a robust health selection process in the attainment of education during adolescence. About 42% of the propensity for school dropout could be attributed to the family level, and is comparable with the results from studies examining years of educational attainment [41]. This underlines the importance of the family as a social context in the process of school dropout and stresses the importance of investigating how health is, or can become, an independent risk factor for school dropout.

### Possible mechanisms

Adolescent health could influence educational attainment through several mechanisms [30]. Poor health could impair cognitive development or affect educational participation because of absenteeism from school, resulting in poorer school achievement [42]. It is also possible that poor health could weaken peer relationships, which could have secondary effects on educational attainment. Additionally, adolescents themselves, parents and teachers could have reduced educational expectations of an adolescent who is limited by poor health. Reduced encouragement and investment in education could also occur if the expected benefits from education (employment) were regarded as low. Youth may also be stigmatized and subsequently discriminated by peers and teachers for some health problems like obesity, which can affect youths motivation and willingness to attend school [31], [43], [44].

### Conclusion and policy implication

Poor health in all its dimensions compromises the opportunity to complete high school for adolescents of all social classes, and the educational gradient that develops with poor health in the picture reduces future work prospects and adult health. Public health policies should ensure that young people with poor health are provided with the best attainable education, thereby preventing them from having their future opportunities substantially reduced. There is still a gap in our information about the mechanisms at work in the relationship between adolescent health and educational attainment. Further research will need to focus on the family perspective, but also a life course perspective in order to better understand adolescents’ social integration process through education. With more knowledge on this topic, additional preventive measures at an early stage may reduce the number of young people living on the fringe of society with poor health and poor prospects for working life.
